# AN EVIDENCE-BASED APPROACH TO TELEPHONE REASSURANCE CALLS: A RANDOMIZED CONTROLLED TRIAL PROTOCOL

**DOI:** 10.1093/geroni/igac059.3011

**Published:** 2022-12-20

**Authors:** Morgan Minyo, David Bass, Catherine McCarthy, Katherine Judge, Fatima Perkins, Douglas Beach

**Affiliations:** Cleveland State University, Cleveland, Ohio, United States; The Benjamin Rose Institute on Aging, Cleveland, Ohio, United States; Benjamin Rose Institute on Aging, Cleveland, Ohio, United States; Cleveland State University, Cleveland, Ohio, United States; Western Reserve Area Agency on Aging, Western Reserve Area Agency on Aging, Ohio, United States; Western Reserve Area Agency on Aging, Western Reserve Area Agency on Aging, Ohio, United States

## Abstract

The COVID-19 Pandemic forced many health and community organizations to suspend some previously offered services and supports for older adults. This created heightened concerns about negative impacts of isolation and loneliness on already vulnerable older adults. In response, the Western Reserve Area Agency on Aging (WRAAA) began the TeleCare Program, providing weekly telephone reassurance calls to home and congregate meal clients to maintain contact and identify unmet service needs. Because of its success, the TeleCare Program has continued as a regularly offered service, but the WRAAA sought to improve the Program by integrating it with BRI Care ConsultationTM, an evidence-based program of the Benjamin Rose Institute on Aging (BRIA). This poster reports on initial results of a randomized controlled trial to test the efficacy and feasibility of a new protocol for delivering telephone reassurance calls. The new protocol is modeled after the Initial Assessment in BRI Care Consultation, which is a semi-structured, telephone conversation covering a comprehensive range of potentials problems. The protocol also connects high-risk clients to existing available home- and community-based services. Results demonstrate that components of proven evidence-based interventions are feasible to integrate into existing programs and services. This includes adapting a model for problem-identification and assessment based on a consumer-driven conceptual framework, a record keeping system to enhance consistency and fidelity of program delivery, and establishing a formal referral protocol to more seamlessly link clients to other available services.

